# PKM2 Drives Hepatocellular Carcinoma Progression by Inducing Immunosuppressive Microenvironment

**DOI:** 10.3389/fimmu.2020.589997

**Published:** 2020-10-20

**Authors:** Tian-En Li, Shun Wang, Xiao-Tian Shen, Ze Zhang, Mo Chen, Hao Wang, Ying Zhu, Da Xu, Bei-Yuan Hu, Ran Wei, Yan Zheng, Qiong-Zhu Dong, Lun-Xiu Qin

**Affiliations:** ^1^ Department of General Surgery, Huashan Hospital & Cancer Metastasis Institute, Fudan University, Shanghai, China; ^2^ Institutes of Biomedical Sciences, Fudan University, Shanghai, China

**Keywords:** hepatocellular carcinoma, pyruvate kinase M2, progression, immune escape, immunosuppressive microenvironment, PD-L1

## Abstract

**Background and Aims:**

Pyruvate kinase M2 (PKM2) is an essential regulator of the Warburg effect, but its biological function promoting immune escape of hepatocellular carcinoma (HCC) is unclear.

**Methods:**

GEPIA web tool and immunohistochemistry (IHC) analysis were employed to evaluate the clinical relevance of PKM2 in HCC patients. Both *in vitro* CCK-8, colony formation, and transwell assays, and *in vivo* xenografts were performed to evaluate the malignancy of HCC cells. PKM2 and PD-L1 levels were examined by Western blot, qRT-PCR, and IHC. The role of PKM2 on *in vivo* immune response was also investigated.

**Results:**

PKM2 was significantly upregulated in HCC and associated with a poor prognosis of HCC patients. Knockdown of PKM2 inhibited *in vitro* proliferation, migration, and invasion of HCC cells, as well as *in vivo* tumor growth. Strikingly, PKM2 showed a strong correlation with the expression of immune inhibitory cytokines and lymphocyte infiltration in HCC. The overexpression of PKM2 sensitized HCC to immune checkpoint blockade, which enhanced IFN-γ positive CD8 T cells in HCC mice models.

**Conclusion:**

PKM2 might be a predictor and a potential therapeutic target for immune checkpoint inhibitors in HCC.

## Introduction

As one of the most common human malignancies, hepatocellular carcinoma (HCC) is the fourth leading cause of cancer-related deaths worldwide (1). Despite the increasing global incidence and status, there has been a dearth of treatment options for advanced HCC. In addition to some multikinase inhibitors such as Lenvatinib and Sorafenib have been proved to be standard treatment, recently, PD-1/PD-L1 blockade therapy have achieved remarkable success. However, their efficacy is limited with less than 20% of patients who would benefit from them (2, 3). Therefore, it is urgent to identify useful biomarkers to select the suitable patients for these therapies.

Previously, our study has shown that glucose metabolic shift influences the HCC progression (4). Cancer cells exhibit aberrant metabolism characterized by shifting energy production from oxidative phosphorylation to glycolysis, known as the “Warburg effect” or “aerobic glycolysis” (5–7). The enhanced glycolysis results in lactic acid accumulation in tumor microenvironment, which helps tumor cells to invade the host’s immunosurveillance and enhances tumor survival (8–11). The relation between immunosurveillance and metabolic reprogramming has been verified. Previous studies found that IL-22, produced by immune cells including T cells and NK cells, could promote oxidative phosphorylation and glycolysis and drive metabolic adaptive reprogramming (12). Pyruvate kinase M2 (PKM2) is considered as an essential regulator of this effect associated with cancer (13). Besides, PKM2 is critical in promoting cancer progression (13, 14). It have been found to phosphorylate essential proteins related to cancer progression and metabolism including Bub3 (15), STAT3 (16), histone H3 (17), and SREB1a (18). It also demonstrated that PKM2 can regulate HIF-1α activity, resulting in the regulate gene expression *via* enhancing its ability to bind to the hypoxia-responsive elements (HREs) (19). Accumulative studies have shown that PKM2 is overexpressed in various cancers including HCC, and targeting PKM2 increases the therapeutic effect of cancer (20–23).

Tumor cells escape from immunosurveillance in a variety of ways (24). PD-L1/PD-1-induced T cell exhaustion is one of the main causes of immune evasion (24–26). Among the variant kinds of cancer immunotherapies, PD-L1/PD-1 blockade therapy has represented the backbone of improving the objective response and survive of patients with cancer (27, 28), including HCC (29, 30). Accumulative studies show that PD-L1 expression has been widely adopted as a predictive biomarker for PD-L1/PD-1 blockade therapy (25, 31). Thus, understanding PD-L1 intrinsic regulatory mechanisms is of great importance for immune checkpoint blockade therapy. It has been reported that PD-L1 expression was driven constitutively by aberrant signaling pathways or chromosomal alterations, including the interferon receptor adapter JAK2 (32, 33), PTEN deletions or PI3K/AKT mutations (34), MYC overexpression (35), CDK5 disruption (36), or increase in PD-L1 transcripts stabilized by truncation of the 3’-untranslated region (UTR) (37). Moreover, PD-L1 up-regulation under hypoxia depended on HIF-1α, and this has been reported to be related with PKM2 as a nuclear-transcription factor (38, 39).

In the present study, we aimed to explore the role of PKM2 in the immune escape of HCC cells, and its potential application in immune therapy of HCC.

## Materials and Methods

### Cells and Animals

HEK 293T cells, human HCC cell lines (HepG2, Hep3B, PLC/RCF/5, and Huh7) and mouse HCC cell line (Hepa1-6) were purchased from the Institute of Biochemistry and Cell Biology, Chinese Academy of Science (Shanghai China). Human HCC cell lines MHCC97-L, MHCC97-H, and HCC-LM3 were obtained from the Liver Cancer Institute at Fudan University (Shanghai, China) as previously described (40, 41). These cell lines were cultured in Dulbecco’s modified Eagle’s Medium (DMEM) (Hyclone) supplemented with 10% fetal bovine serum (FBS) (Gibco). All cell lines were routinely maintained at 37.0°C in a humidified incubator with 5% CO_2_.

All male mice (C57BL/6, BALB/c nu/nu) were purchased from Shanghai Slac Laboratory Animal Co. and maintained on standard rodent chow and water ad libitum. Prior to any surgical procedures, all mice were given intraperitoneal (i.p.) injection of Pentobarbital Sodium (40 mg/kg; Sigma Aldrich, St.Louis, MO, USA) and conducted in the SPF laboratory. All procedures involving animals were approved by The Animal Care and Use Committee of Fudan University (Shanghai, China).

### Establishment of HCC Subcutaneous Xenograft Models in Nude Mice

Four human HCC cells including Sh-control MHCC97-H, Sh-PKM2 MHCC97-H, OE-vector Huh7 and OE-PKM2 Huh7 (2 × 10^6^/100 μl in 50% Matrigel and PBS per mice) were injected into the right flank of nude mice (5 mice/group) to establish the subcutaneous implantation models. The tumor growth was monitored once per week until the mice were sacrificed. Tumor volume was calculated by the formula: a × b^2^/2 (a and b represent the largest and smallest tumor diameters respectively). The tumors were collected for RNA preparation, fixed in 4% formalin, and then embedded in paraffin. Consecutive sections of tumor tissues were applied by immunohistochemistry (IHC) staining.

Four mouse HCC cells including Sh-control Hepa1-6, Sh-PKM2 Hepa1-6, OE-control Hepa1-6, and OE-PKM2 Hepa1-6 (1 × 10^6^/100 μl in 50% Matrigel and PBS per mice) were injected into the right flank of C57BL/6 mice (5 mice/group). All the tumors were collected, and the tumor weight was measured.

## Results

### PKM2 Is Up-Regulated in HCC and Associated With Poor Prognosis in HCC Patients

**Figure 1:**
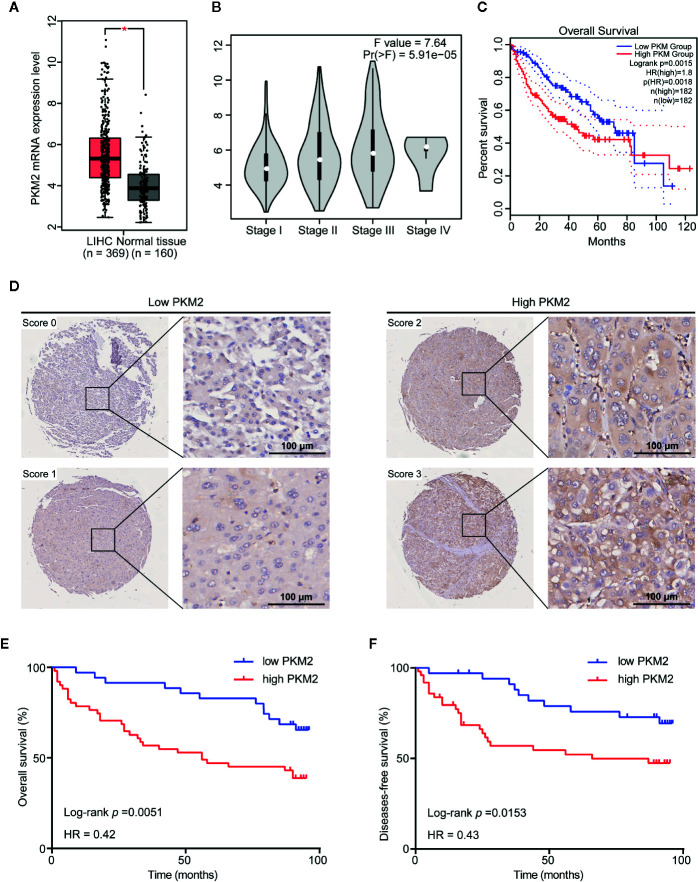
PKM2 is upregulated in HCC and associated with poor prognosis in HCC patients. **(A)** The GEPIA database revealed that PKM2 expression was significantly upregulated in HCC patients. The boxplot analysis showed log2 (TPM+1) on a log-scale. **(B)** Correlation between the expression level of PKM2 and the pathological stage of HCC patients (GEPIA). **(C)** The overall survival (OS) of the patients with HCC was computed with the GEPIA web tool. **(D)** Representative images of IHC staining of PKM2 in HCC tissue microarrays. Scale bar: 100 µm. **(E**, **F)** Survival curves of OS **(E)** and disease-free survival (DFS) **(F)** in HCC patients with differential PKM2 expression level calculated by Kaplan-Meier analysis and compared with the Log-rank test.

To investigate the role of PKM2 in malignant progression of HCC, we used GEPIA web tool to analyze PKM2 mRNA expression in HCC and nontumor liver tissues. The results showed that the mRNA levels of PKM2 in HCC tissues were significantly higher than that in paraneoplastic tissues (**Figure 1A**). In addition, the correlation analysis showed a significant correlation between the expression of PKM2 and the pathological stage (*P* = 5.91e−05) (**Figure 1B**). PKM2 expression was significantly higher in advanced stages of HCCs than that in early ones. The survival curves showed that a higher PKM2 level was significantly associated with poor prognosis of HCC patients (**Figure 1C**).

We further evaluated the relationship between the protein level of PKM2 and clinicopathological features of HCC patients (**Supplementary Table 1**). PKM2 expression was found in significant correlation with HCC tumor size (*P* = 0.013) and tumor grade (*P* = 0.035) rather than the other clinicopathological features including gender, age, HBV status, liver cirrhosis, AFP, ALT, tumor numbers, tumor capsule, and BCLC stage (**Supplementary Table 2**). Survival analysis showed that patients with high PKM2 expression had shorter overall survival (OS) and disease-free survival (DFS) time (log rank *P* = 0.0153 and 0.0051, respectively; **Figures 1D–F**).

### PKM2 Promotes HCC Progression

**Figure 2:**
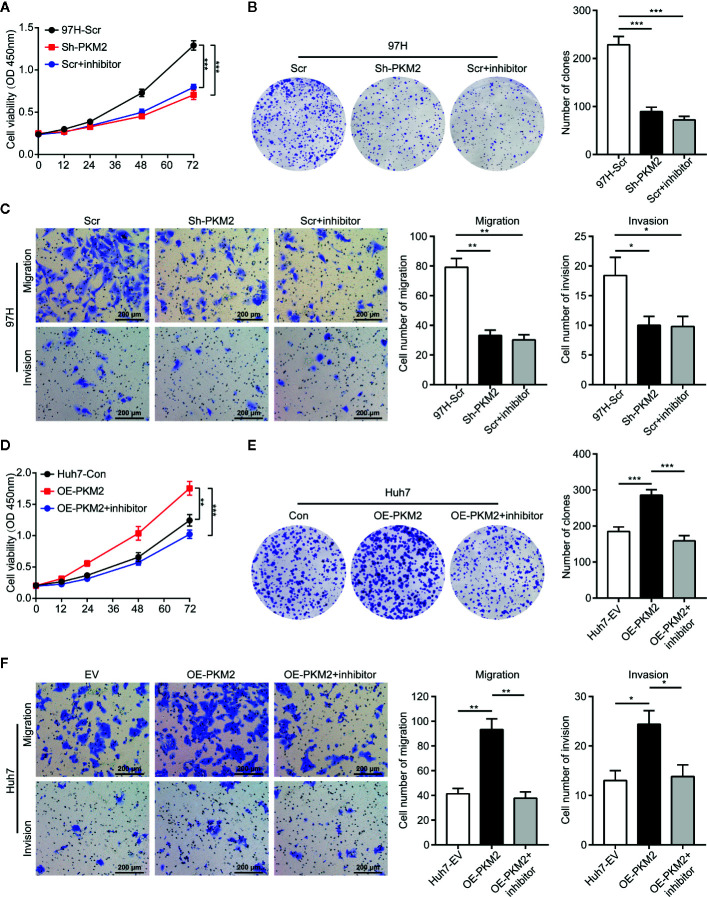
PKM2 promotes cell proliferation, invasion and migration of HCC cells *in vitro*. **(A)** Cell proliferations of MHCC97-H cells with Sh-control and Sh-PKM2 were assessed by CCK-8 assays. **(B)** Images (left panel) and quantified analysis (right panel) of clone formation assays in MHCC97-H cells with Sh-control, Sh-PKM2 and PKM2 inhibitor. **(C)** Representative images (left panel) and quantified analysis (right 2 panels) of transwell assays in MHCC97-H cells with Sh-control, Sh-PKM2, and PKM2 inhibitor. **(D)** Cell proliferations of OE-vector and OE-PKM2 were assessed by CCK-8 assays in Huh7 cells. **(E)** Images (left panel) and quantified analysis (right panel) of clone formation assays in Huh7 cells with OE-vector and OE-PKM2. **(F)** Representative images (left panel) and quantified analysis (right 2 panels) of transwell assays in Huh7 cells with OE-vector and OE-PKM2. n = 5, mean ± SEM, ***P < 0.001, **P < 0.01, *P < 0.05. Sh, short hairpin; Scr, Sh-control; EV, Overexpression vector; OE, Overexpression; OD, absorbance degrees.

To demonstrate the specific role of PKM2 in HCC cells, we detected expression of PKM2 in HCC cell lines including HepG2, Hep3B, PLC/RCF/5 (PLC), Huh7, MHCC97-L, MHCC97-H and HCC-LM3 (**Supplementary Figure 1A**). The results indicated that PKM2 was more highly expressed in HCC cell lines with high metastasis potential including MHCC97-H and HCC-LM3 compared with the others including HepG2, Hep3B, PLC, and Huh7. Then, Knockdown of PKM2 with specific lentiviral short hairpin RNA (Sh-PKM2) (**Supplementary Figures 2A, B**) or PKM2 inhibitor significantly impeded the cell proliferation of MHCC97-H cells with high intrinsic PKM2 overexpression (**Figure 2A**). Likewise, the numbers of clone formation (**Figure 2B**), as well as the migration and invasion capacities (**Figure 2C**) were notably decreased in MHCC97-H cells with Sh-PKM2 or treated with PKM2 inhibitor. On the other hand, up-regulation of PKM2 by transfection of expression plasmid of PKM2 (OE-PKM2 Huh7 cells) (**Supplementary Figures 2C, D**) significantly enhanced proliferation, migration and invasion capacities of Huh7 cells, which have a low intrinsic PKM2 level (**Figures 2D–F**). These indicate that PKM2 plays important roles in promoting *in vitro* proliferation and invasion of HCC cells.

**Figure 3:**
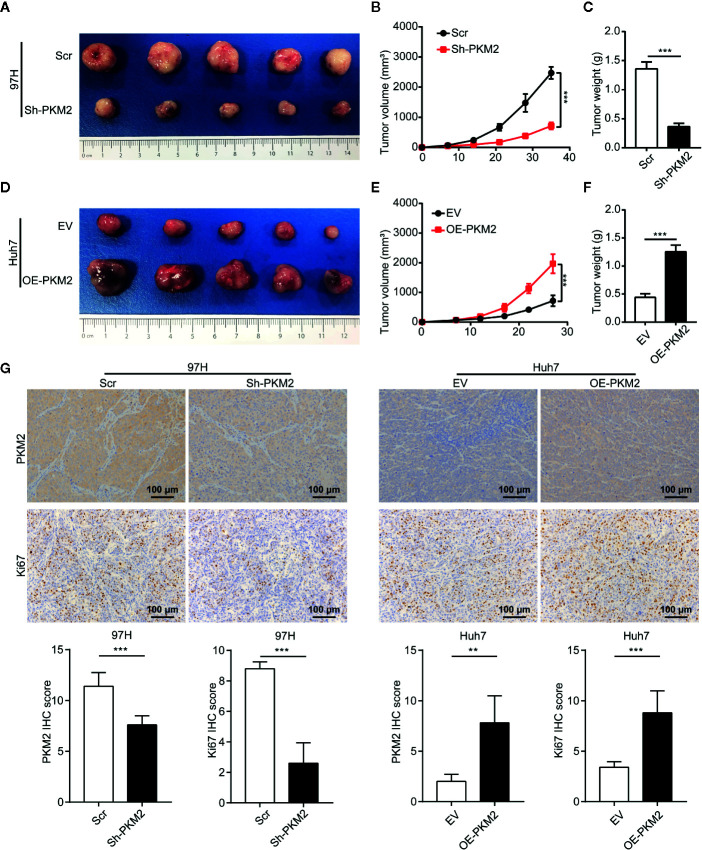
PKM2 promotes HCC proliferation *in vivo*. **(A–C)** The final representative images **(A)**, tumor growth **(B)** and tumor weight **(C)** of xenograft tumors from MHCC97-H with Sh-control and Sh-PKM2. **(D–F)** The final representative images **(D)**, tumor growth **(E)**, and tumor weight **(F)** of xenograft tumors from Huh7 with OE-vector and OE-PKM2. **(G)** The representative imagines and quantitative analysis of IHC staining of PKM2 and Ki67 in Sh-PKM2 MHCC97-H xenograft tumors and OE-PKM2 Huh7 xenograft tumors. Upper panel: representative imagines. Lower panel: quantitative analysis. Scale bar: 100 µm. n = 5, mean ± SEM, ***P < 0.001, **P < 0.01.

To validate the role of PKM2 *in vivo*, we established subcutaneous xenograft models using various HCC cells. Knockdown of PKM2 significantly inhibited the tumor growth of MHCC97-H cells, and the tumors of Sh-PKM2 group were significantly smaller than that of the Sh-controls (**Figures 3A–C**). On the other hand, overexpression PKM2 significantly promoted the tumor growth of Huh7 cells, and the tumors in the OE-PKM2 group were significantly larger than the controls (**Figures 3D–F**). In addition, IHC staining of PKM2 and Ki67 in xenografts tumors showed that knockdown of PKM2 inhibited proliferation of MHCC97-H tumors, while overexpression of PKM2 promoted the proliferation of Huh7 tumors *in vivo* (**Figure 3G**). These provide further evidence that PKM2 promotes tumor growth and metastasis of HCCs.

### PKM2 Modulates Glucose Metabolic Reprogramming in HCC

**Figure 4:**
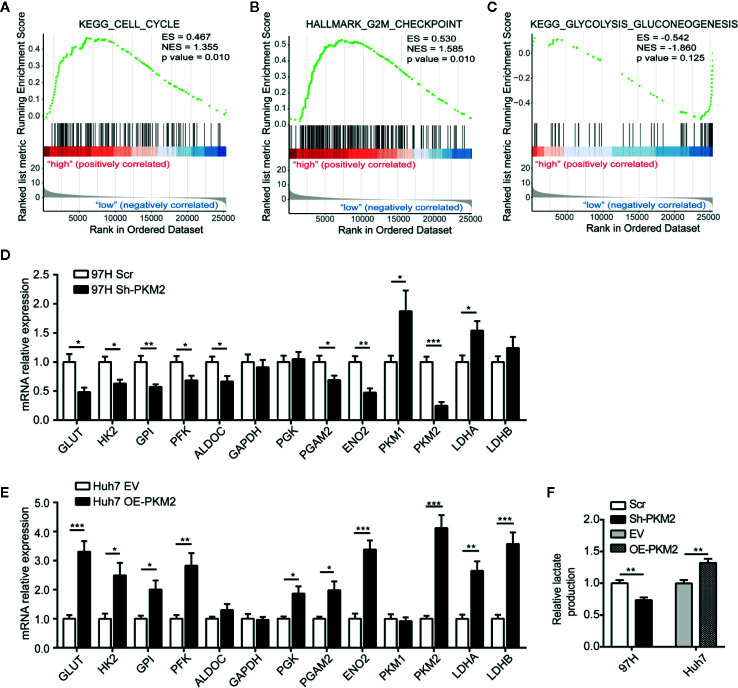
PKM2 regulates cell cycle and metabolic reprogramming of HCC cells. **(A–C)** GSEA analysis of TCGA data showing the enriched hallmark signatures involved in Cell Cycle **(A)**, G2M checkpoint **(B)** and Glycolysis-Gluconeogenesis **(C)** in HCC with high PKM2 expression. **(D**, **E)** Aerobic glycolysis-related enzymes detected using qRT-PCR in sh-PKM2 MHCC97-H cells **(D)** and OE-PKM2 Huh7 cells **(E)**. **(F)** Relative lactate production in Sh-PKM2 MHCC97-H cells and OE-PKM2 Huh7 cells. n = 5, mean ± SEM, ***P < 0.001, **P < 0.01, *P < 0.05.

To investigate the mechanism of PKM2 regulating HCC malignancy potential, we performed Gene Set Enrichment Analysis (GSEA) from TCGA data. Compared with the PKM2 low expression group, the leading enriched signatures in PKM2 high expression group included gene sets involved in cell cycle (**Figure 4A**), G2/M checkpoint (**Figure 4B**), and glycolysis-gluconeogenesis (**Figure 4C**). To verify the metabolic shift induced by PKM2 alteration in HCC, we used qRT-PCR to detect the expression of metabolic enzymes in glucose metabolism. The representative enzymes involved in aerobic glycolysis were significantly reduced in Sh-PKM2 MHCC 97-H cells (**Figure 4D**) and their subcutaneous xenograft tumors (**Supplementary Figure 3A**), while the expression levels of key metabolic enzymes were significantly increased in OE-PKM2 Huh7 cells (**Figure 4E**) and their subcutaneous xenograft tumors (**Supplementary Figure 3B**). Moreover, knockdown of PKM2 effectively inhibited lactate production of MHCC97-H cells and overexpression of PKM2 enhanced lactate production in Huh7 cells (**Figure 4F**). These indicate that PKM2 promotes the progression of HCC *via* regulating glucose metabolism.

### PKM2 Remodels Tumor Immune Microenvironment in HCCs

**Figure 5:**
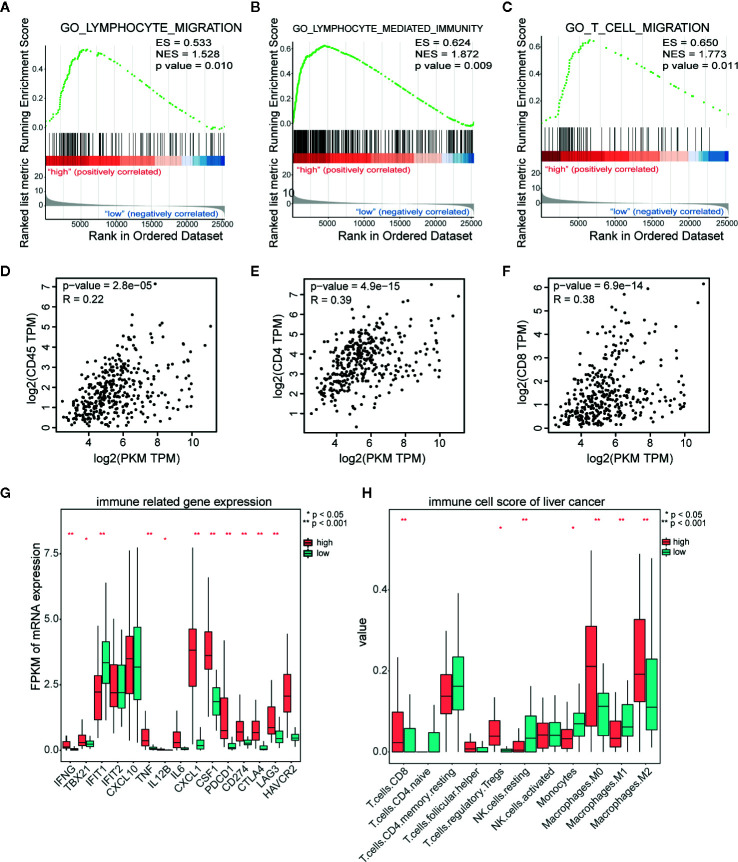
PKM2 expression is in distinct correlation with lymphocyte infiltration and immune inhibitory molecular related gene expression in HCC samples. **(A–C)** GSEA analysis of TCGA data showing the significantly enriched hallmark signatures involved in lymphocyte migration **(A)**, lymphocyte mediated immunity **(B)**, and T cell migration **(C)** in HCC with high PKM2 expression. **(D–F)** The correlations between the mRNA expression levels of PKM2 and CD45 **(D)**, CD4 **(E)**, and CD8 **(F)** in HCC samples determined by the GEPIA web tool. **(G)** Immune related gene expression in human HCC samples with high and low expression of PKM2. **(H)** Various immune cell scores of HCC samples in high and low PKM2 expression.

To explore the role of PKM2 in immune response of HCC microenvironment, we analyzed the lymphocyte infiltration and expression of immune inhibitory genes in TCGA database using GSEA. The most significantly enriched signatures were gene sets involved in lymphocyte migration (**Figure 5A**), lymphocyte mediated immunity (**Figure 5B**) and T cell migration (**Figure 5C**) in PKM2-high group compared with the PKM2-low ones. Then we used the GEPIA web tool to analyze the correlation between PKM2 level and CD45, CD4, or CD8. The results indicated that PKM2 expression was in distinctive correlation with CD45, CD4, and CD8 expression in HCC specimens (**Figures 5D–F**). We further conducted KEGG enrichment analysis to figure out the differential immune-related genes between the HCCs with different PKM2 levels. The tumors with high PKM2 were found to have higher levels of inflammatory factors, such as TNF and IL-6, and chemokines including CXCL1 and CSF1, as well as the immune inhibitory factors, such as CD274 (PD-L1), CTLA4, and LAG3 (**Figure 5G**). In addition, increased CD8 T cells, Treg cells, and M2 macrophages but decreased M1 macrophages were found in HCCs with high PKM2 (**Figure 5H**). These indicate that the high PKM2 expression induces an inflammatory milieu and creates an immunosuppressive microenvironment to support HCC progression.

### PKM2 Is Positively Correlated With PD-L1 in HCC Patients

**Figure 6:**
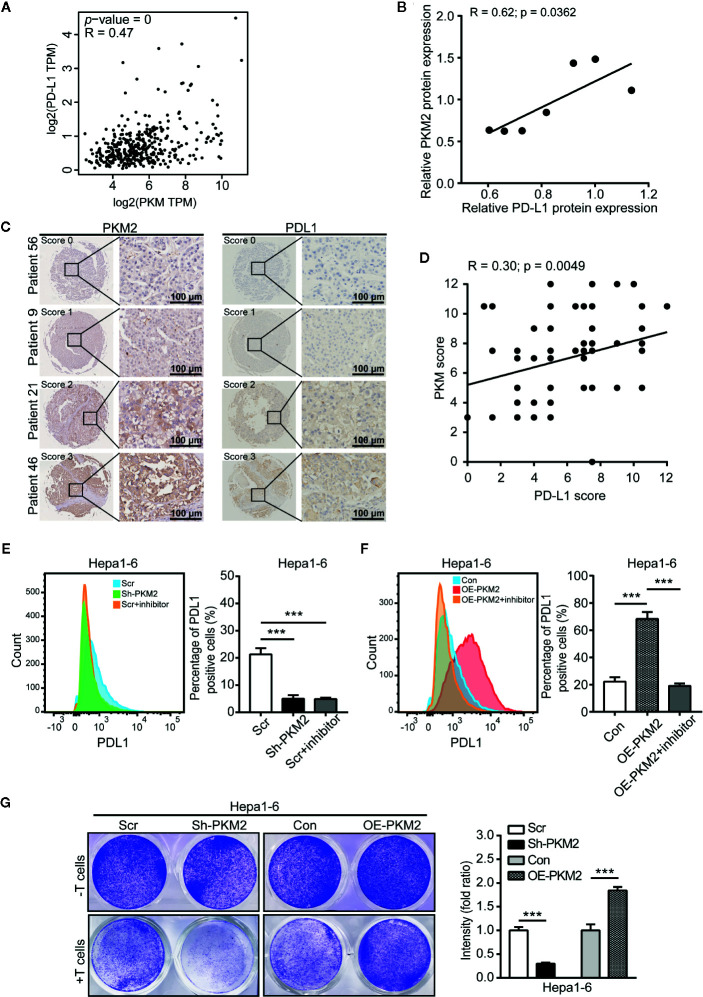
PKM2 promote the immune escape of HCC *via* PD-L1 upregulation. **(A)** The correlation between the expression of PKM2 and PD-L1 in human HCC samples using the GEPIA web tool. **(B)** The correlation between PD-L1 and PKM2 expression in human HCC cell lines. Pearson product-moment correlation coefficients and the *P* values are shown. **(C)** Representative IHC staining of HCC tumors for PKM2 and PD-L1. Scale bar: 100 µm. **(D)** The correlation between the PD-L1 and PKM2 expression in tumor tissues of the HCC patients (n = 87). Pearson product-moment correlation coefficients and the *P* values are shown. **(E)** Flow cytometric analysis and quantitative analysis of PD-L1 expression in Sh-PKM2 Hepa1-6 cells. Left panel: Representative image of flow cytometric analysis. Right panel: Quantitative analysis of flow cytometric analysis. **(F)** Flow cytometric analysis and quantitative analysis of PD-L1 expression in OE-PKM2 Hepa1-6 cells. Left panel: Representative image of flow cytometric analysis. Right panel: Quantitative analysis of flow cytometric analysis. **(G)** Representative images (left panel) and statistical analysis (right panel) of T cell cytotoxicity assays. n = 5, mean ± SEM, ***P < 0.001.

We further used GEPIA to analyze the correlation between PKM2 and PD-L1 expression levels, and found a close correlation between them in HCCs (**Figure 6A**). This correlation was further validated in protein level in HCC cell lines including HepG2, Hep3B, PLC, Huh7, MHCC97-L, MHCC97-H, and HCC-LM3 detected by Western blot (**Figure 6B** and **Supplementary Figure 1A**) and HCC specimens using IHC on the TMA (**Figure 6C**). The association of PD-L1 expression with clinicopathological parameters was summarized in **Supplementary Table 3**. PD-L1 expression was associated with tumor size (*P* = 0.026) and tumor number (*P* = 0.08), rather than the other clinicopathological features. Pearson product-moment correlation analysis showed a significantly positive correlation between PD-L1 and PKM2 (**Figure 6D**, r = 0.30, *P* = 0.0049).

To further determine this correlation, we detected the PD-L1 level in HCC cells after PKM2 expression were modulated. Both the mRNA and protein levels of PD-L1 were significantly decreased when PKM2 was knocked down by Sh-PKM2 in MHCC97-H cells (**Supplementary Figure 4A**) but significantly increased in OE-PKM2 Huh7 cells (**Supplementary Figure 4B**). Similar alterations were observed in Hepa1-6 cells (**Supplementary Figure 3C, D**), as well as in the xenograft tumors of Sh-PKM2 MhCC97-H and OE-PKM2 Huh7 cells (**Supplementary Figure 4B**). Flow cytometric (FCM) analysis validated the decreasing expression of PD-L1 in MHCC97-H cells and Hepa1-6 cells after PKM2 knockdown or PKM2 inhibitor treatment, whereas overexpression of PKM2 increased PD-L1 expression in Huh7 and Hepa1-6 cells (**Figures 6E, F** and **Supplementary Figures 6A, B**). Moreover, Sh-PKM2 transfected Hepa1-6 cells were more sensitive to cytotoxicity mediated by CD8 T cell than the control Hepa1-6 cells, whereas OE-PKM2 Hepa1-6 cells were more resistant to CD8 T cell mediated cytotoxicity than the controls (**Figure 6G**). These results indicate that PKM2 is closely correlated to PD-L1 expression and immune escape in HCCs and may serve as a biomarker for the response to PD-L1 antibody treatment.

### Overexpression of PKM2 Enhances the Therapeutic Response of HCC to PD-L1 Blockade

**Figure 7:**
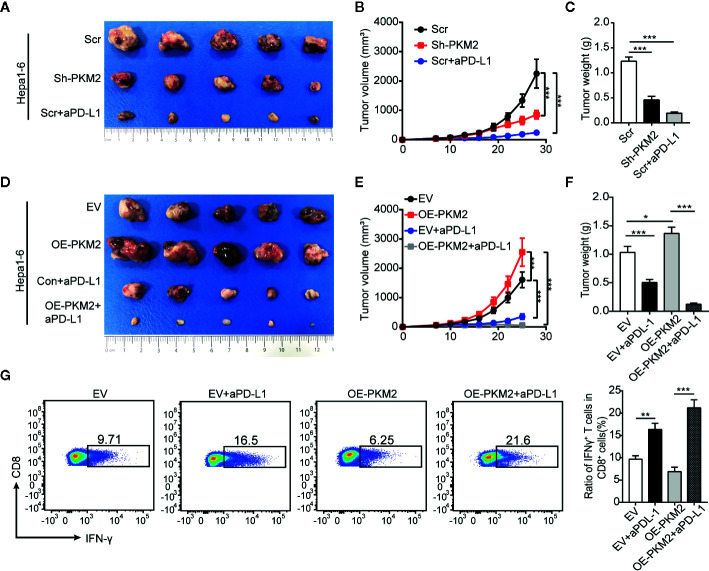
PKM2 sensitizes Hepa1-6 tumors to anti-PD-L1 antibody. **(A–C)** The final representative images **(A)**, tumor growth **(B)** and tumor mass **(C)** of xenograft Hepa1-6 tumors from Sh-control, Sh-PKM2 and Scr-aPD-L1 groups. **(D** and **E)** The final representative images **(D)**, tumor growth **(E)** and tumor mass **(F)** of xenograft Hepa1-6 tumors from OE-vector, OE-PKM2, EV-aPD-L1, and OE-PKM2-PD-L1 group. **(G)** Flow cytometric analysis (left panel) and statistical analysis (right panel) of IFN-γ secretion of CD8 T cells from each group. n = 5, mean ± SEM, ***P < 0.001, **P < 0.01, *P < 0.05.

In addition to PKM2 as a biomarker for PD-L1 blockade therapy, we further explored the effect of PKM2 level on the response of PD-L1 blockade. We treated the syngeneic C57BL/6 mice bearing with Sh-PKM2 Hepa1-6 cells or the Sh-control cells with a murine anti-PD-L1 antibody (aPD-L1) at first. Either PKM2 knockdown or aPD-L1 therapy could significantly inhibit *in vivo* tumor growth compared with the controls **(** **Figures 7A–C**). Besides, we treated OE-PKM2 Hepa1-6 and the OE-vector Hepa1-6 bearing mice with aPD-L1 to elucidate the PKM2 overexpression on the response to PD-L1 blockade. OE-PKM2 was found to significantly enhance the inhibitory effect of aPD-L1, although aPD-L1 alone also suppressed the tumor growth in subcutaneous implantation models (**Figures 7D–F**). The increase of IFN-γ secretion induced by aPD-L1 therapy was also much more significantly in the mice from OE-PKM2 group than the OE-vector group (**Figure 7G**). Taken together, these results indicate that PKM2 promotes PD-L1 expression and enhances the response of HCC to PD-L1 blockade therapy.

## Discussion

HCC is the fourth leading cause of cancer related deaths worldwide, but currently there are no effective targeted therapies for the intermediate and advanced HCC (1, 42, 43). In addition to the traditional antiangiogenic tyrosine kinase inhibitors, anti-PD-L1 and anti-PD-1 monoclonal antibodies as well as combined therapies (30, 44) have shown promising antitumor effects, but only a fraction of treated patients achieves durable responses (2, 3, 43–45). These suitable patients of efficient therapeutic strategies need to be selected by specific biomarkers. Accumulative works have demonstrated the key role of metabolic enzymes in tumor microenvironment (46–48). Molecular typing and targeted interventions which based on metabolic reprogramming have become potential therapeutic strategies for HCC (49, 50). Herein, we report that PKM2 drives HCC progression by inducing an immunosuppressive microenvironment. The overexpression of PKM2 enhances the therapeutic response of HCC to PD-L1 blockade.

PKM2 is an essential regulator of the Warburg effect in cancer cells which catalyzes the phosphoenolpyruvate (PEP) and adenosine diphosphate (ADP) into pyruvate and adenosine triphosphate (ATP) as fuel for proliferation and metastasis of cancer cells (51). Accumulative studies have reported that PKM2 is upregulated and promotes the growth and metastasis in multiple tumors (52, 53). In the present study, we found that PKM2 was highly expressed in HCCs and was closely correlated to the poor prognosis of HCC patients. PKM2 was proved to promote HCC growth and metastasis *via in vitro* and *in vivo* assays. Our findings reinforce the idea that metabolic enzyme such as PKM2 is an important mediator for HCC progression. Because HCC are well known to be highly aggressive tumors, it appears that PKM2 may serve as a potential metabolic biomarker for prediction of HCC prognosis, and an effective treatment target to block HCC progression.

Growing evidence indicated that metabolic abnormalities promotes immune escape of cancer cells. Glucose consumption by cancer cells metabolically restricts T cells *via* dampened their glycolytic capacity and IFN-γ production (54). Abnormal accumulation of lactic acid due to excessive glycolysis and further acidic environment lead to cytotoxic effects on various immune cells (55). On the other hand, aerobic glycolysis orchestrates a molecular network of chemokines to affect myeloid-derived suppressor cells (MDSCs) and maintains tumor immunosuppression (46). In this study, we found PKM2 was in a distinctive correlation with immunosuppressive microenvironment of HCC. Although CD45, CD4 and CD8 immune cells were abundantly infiltrated in the tumor microenvironment of HCC patients with high level PKM2, these patients also have higher levels of inflammatory factors, such as TNF and IL-6, and chemokines including CXCL1 and CSF1, as well as the immune inhibitory factors, such as CD274 (PD-L1), CTLA4, and LAG3. Moreover, Treg cells and M2 macrophages were increasingly infiltrated, while M1 macrophages were decreasingly infiltrated in the high PKM2 tumor microenvironment. These alterations of immune cells and immune factors are responsible for immune escape, which, in turn, promote the progression of HCC. These findings further underscore the close relationship between metabolic alterations and immune responses.

In addition, we found that PKM2 expression was positively correlated with regulators of PD-L1 includes inflammatory signaling like IFN-γ and oncogenic signaling such as MYC, HIF1α and STATA3 (56). As PKM2 promotes HIF1a downstream gene transcription through direct interaction with HIF1α (19), which may be one of the mechanisms why PKM2 promotes PD-L1 expression. On the other hand, our previous study found that CSF1-induced macrophage polarization toward M2 reversely promoted PD-L1 expression of tumor cell *via* PI3K/AKT/NF-κB/p65 activation (40). It may uncover a new insight on the PD-L1 regulation *via* interaction of tumor cells and immune cells.

The discovery of biomarkers that predict immunotherapeutic response is essential for the clinical application of anti-PD-1/PD-L1 antibodies. Despite the improved response rates of the patients with higher levels of PD-L1 expression, interpretation of PD-L1 by IHC cannot reflect the true expression of PD-L1 expression. The lack of binding sites on PD-L1 amenable for IHC detection, the variation of IHC cutoffs, and varying detection antibodies contribute to the marred interpretation. besides the PD-L1 expression heterogeneity (57). Thus, the biomarker chaperones are needed to improve the detection accuracy of PD-L1 expression. Our findings demonstrated that high PKM2 expression induces an inflammatory milieu and creates an immunosuppressive microenvironment. This prompted us to hypothesize that anti-PD-1/PD-L1 antibodies were more effective in HCC patients with high PKM2 expression. And it was confirmed by the enhanced therapeutic response to PD-L1 blockade therapy caused by the overexpression of PKM2. These results indicated that PKM2 would promise a probable candidate for improved prediction on PD-L1/PD-1 blockade benefit for HCC patients.

In conclusion, our findings indicated that PKM2 drove HCC progression *via* inducing immunosuppressive microenvironment and PD-L1 upregulation. The overexpression of PKM2 sensitized HCC to immune checkpoint blockade, which enhance IFN-γ positive CD8 T cells in HCC mice models. PKM2 might be a predictor and a potential therapeutic target for immune checkpoint inhibitors in HCC.

## Data Availability Statement

The datasets analyzed for this study can be found in the Gene Expression Profiling Interactive Analysis 2 (GEPIA2) http://gepia2.cancer-pku.cn.

## Ethics Statement

The studies involving human participants were reviewed and approved by Ethics Boards of Huashan Hospital of Fudan University (Shanghai, China). The patients/participants provided their written informed consent to participate in this study. The animal study was reviewed and approved by Ethical Committee of Fudan University (Shanghai, China). Written informed consent was obtained from the individual(s) for the publication of any potentially identifiable images or data included in this article.

## Author Contributions

Q-ZD, L-XQ, and T-EL designed the research. SW, T-EL, YiZ, and X-TS performed the research. SW, T-EL, X-TS, ZZ, MC, RW, and HW analyzed the data. T-EL and SW drafted the paper. Q-ZD, DX, and YaZ provided patient tissue samples and collected patient clinical information. Q-ZD, L-XQ, and T-EL revised the paper. All authors contributed to the article and approved the submitted version.

## Funding

This work was supported by the Program of Shanghai Academic Research Leader (20XD1400900), the National Key Research and Development Program of China (2017YFC1308604 and 2017YFC0908402), the National Natural Science Foundation of China (81940074, 81772563, 81802903, and 81672820), the NSFC Program of International Cooperation and Exchanges (81120108016).

## Conflict of Interest

The authors declare that the research was conducted in the absence of any commercial or financial relationships that could be construed as a potential conflict of interest.
