# Supplementary material for: PKM2 Drives Hepatocellular Carcinoma Progression by Inducing Immunosuppressive Microenvironment
Source: Front Immunol. 2020 Oct 20;11:589997. doi: 10.3389/fimmu.2020.589997 (PMC7606949; doi:10.3389/fimmu.2020.589997)
Supplement: Supplementary file 1 [file Table_1.docx]

Supplementary Figures

## Supplementary Figure 1


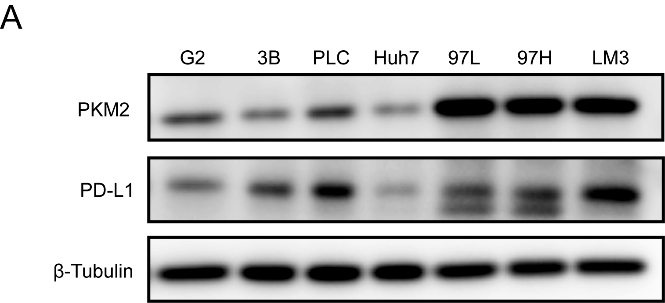


**Supplementary Figure 1 PKM2 and PD-L1 expression detected by western blot in human HCC cell lines.** (A) PKM2 and PD-L1 expression detected by western blot in human HCC cell lines.

## Supplementary Figure 2


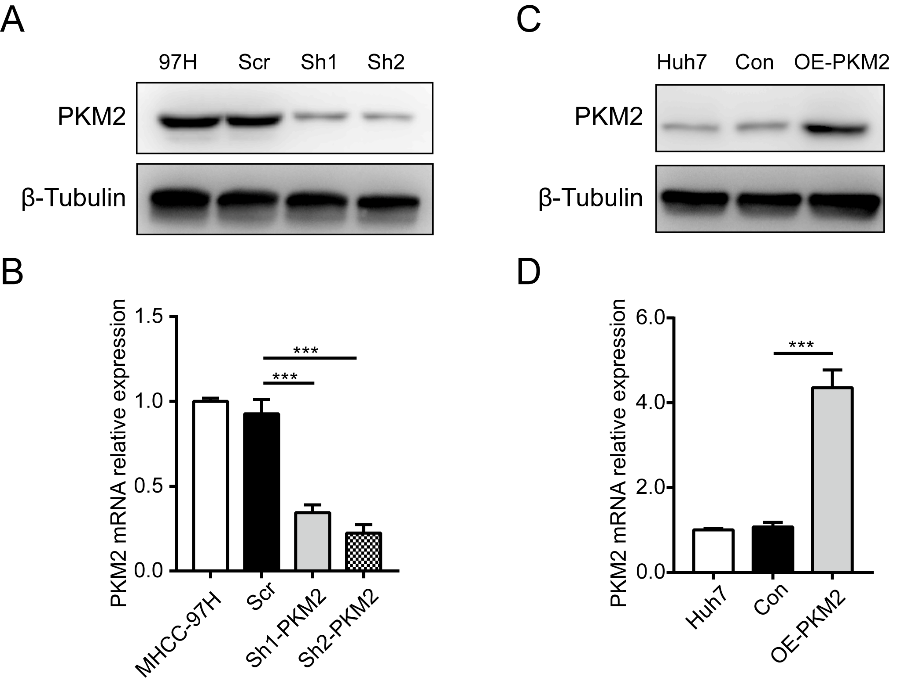


**Supplementary Figure2.** **Efficiencies of the knockdown and overexpression of PKM2.** (A) Western blot confirmed the efficiencies of knockdown PKM2 in 97H cells. (B) qRT-PCR analysis confirmed the efficiencies of knockdown PKM2 in 97H cells. (C) Western blot confirmed the efficiencies of overexpression PKM2 in Huh7 cells. (D) qRT-PCR analysis confirmed the efficiencies of overexpression PKM2 in Huh7 cells.

## Supplementary Figure 3


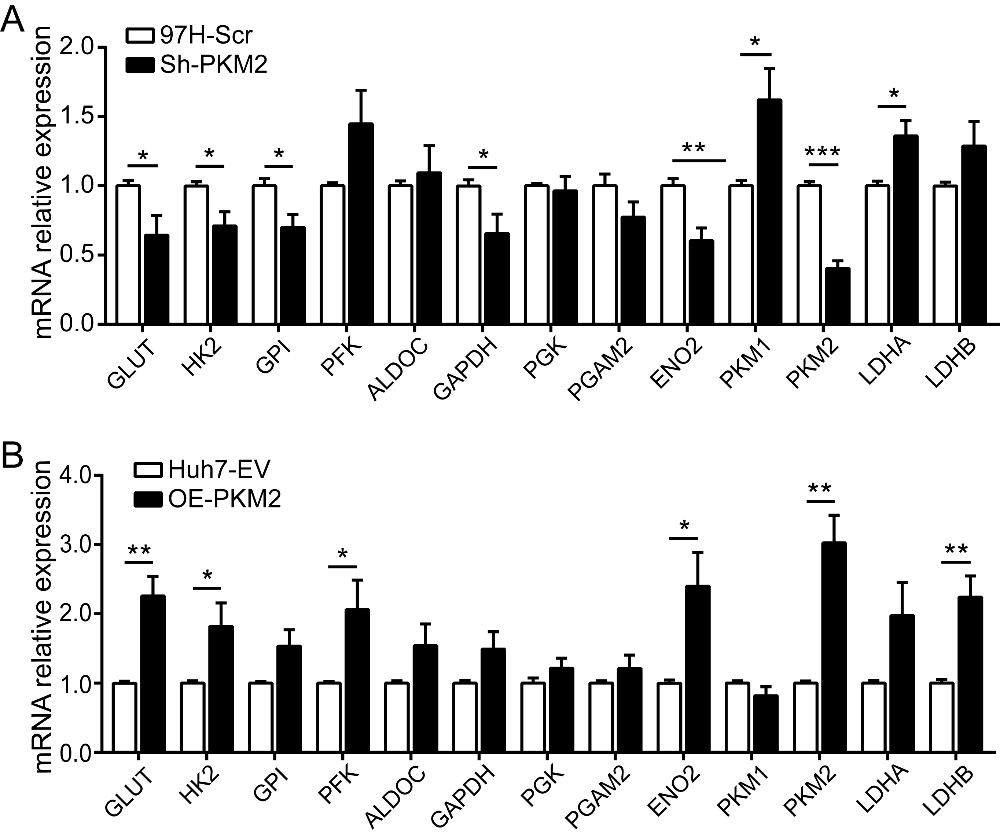


**Supplementary Figure 3.** **PKM2 mediates metabolic reprogramming of HCC cells.** (A) Aerobic glycolysis related enzymes were detected using qRT-PCR in Sh-control and Sh-PKM2 97H subcutaneous tumors. (B) Aerobic glycolysis related enzymes were detected using qRT-PCR in OE-vector and OE-PKM2 Huh7 subcutaneous tumors. mean ± SEM, ***P<0.001, **P< 0.01, *P <0.05.

## Supplementary Figure 4


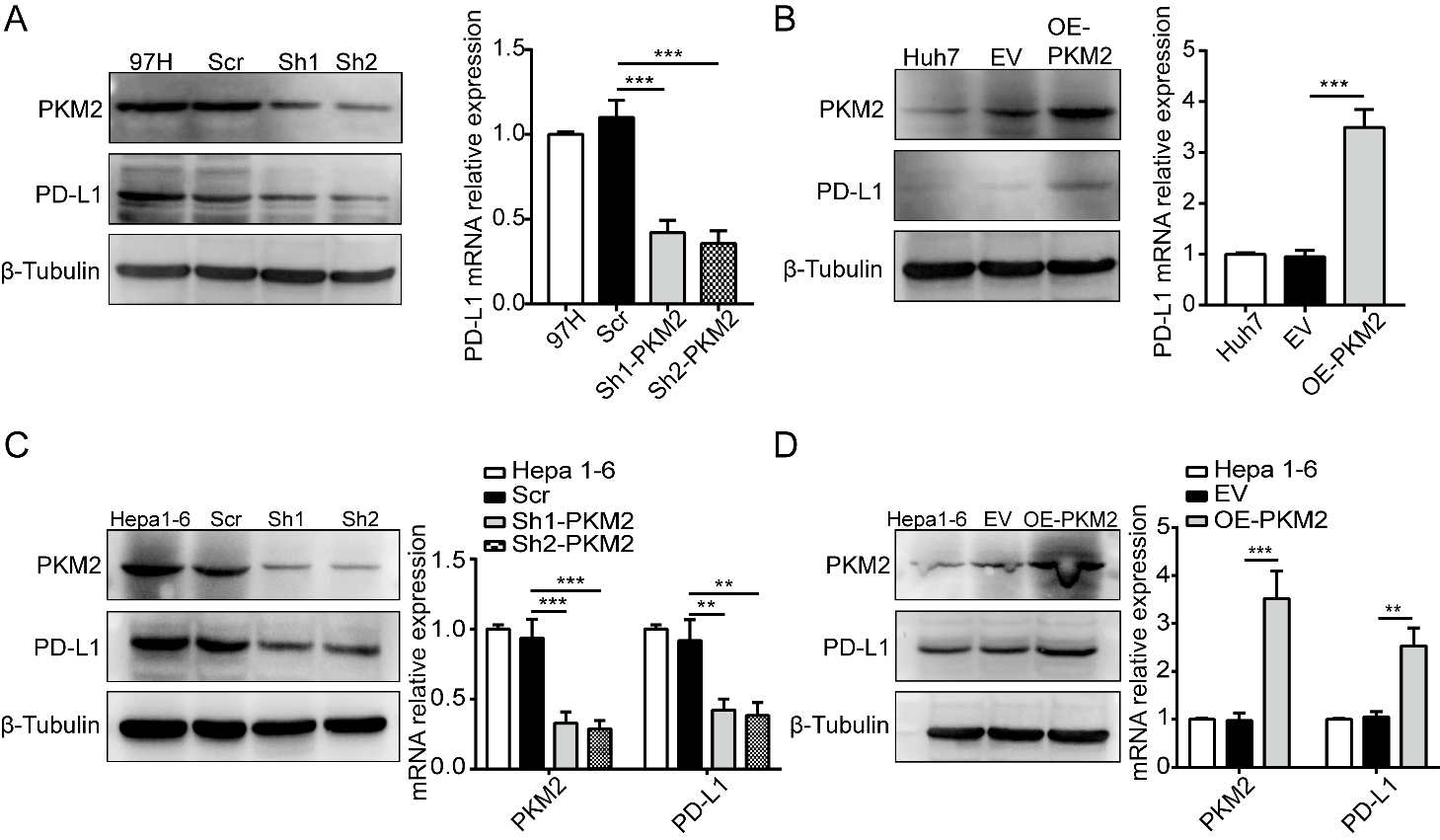


**Supplementary Figure 4.** **The correlation between PKM2 and PD-L1 expression in human and mouse HCC cell lines.** (A) Western blot (left panel) and qRT-PCR (right panel) analysis confirmed the decreased expression of PD-L1 in 97H, Sh-control 97H, Sh1-PKM2, Sh2-PKM2 97H cells. (B) Western blot (left panel) and qRT-PCR (right panel) analysis confirmed the increased expression of PD-L1 in Huh7, OE-vector Huh7 and OE-PKM2 Huh7 cells. (C) Western blot (left panel) and qRT-PCR (right panel) analysis confirmed the decreased expression of PD-L1 in Hepa1-6, Sh-control Hepa1-6, Sh1-PKM2 Hepa1-6, Sh2-PKM2 Hepa1-6 cells. (D) Western blot (left panel) and qRT-PCR (right panel) analysis confirmed the increased expression of PD-L1 in Hepa1-6, OE-vector Hepa1-6 and OE-PKM2 Hepa1-6 cells.

**Supplementary Figure 5**


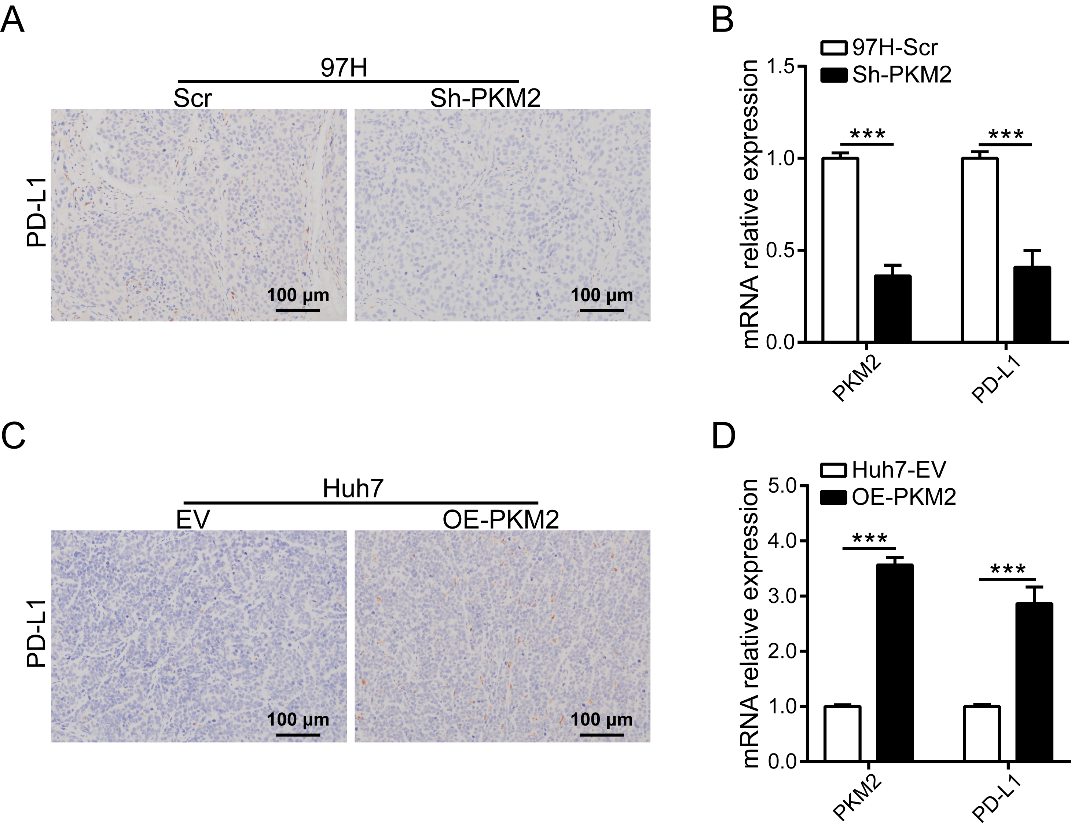


**Supplementary Figure 5.** **PD-L1 expression in control and PKM2 intervened MHCC97-H and Huh7 subcutaneous tumors.** (A) The representative imagines of IHC staining of PD-L1 in Sh-control and Sh-PKM2 97H xenograft tumors. (B) PKM2 and PD-L1 mRNA levels were detected using qRT-PCR in Sh-control and Sh-PKM2 97H xenograft tumors. (C) The representative imagines of IHC staining of PD-L1 in OE-vector and OE-PKM2 Huh7 xenograft tumors. (D) PKM2 and PD-L1 mRNA levels were detected using qRT-PCR in OE-vector and OE-PKM2 Huh7 xenograft tumors. Scale bar: 100 µm. n= 5, mean ± SEM, ***P<0.001, **P< 0.01, *P <0.05.

**Supplementary Figure 6**


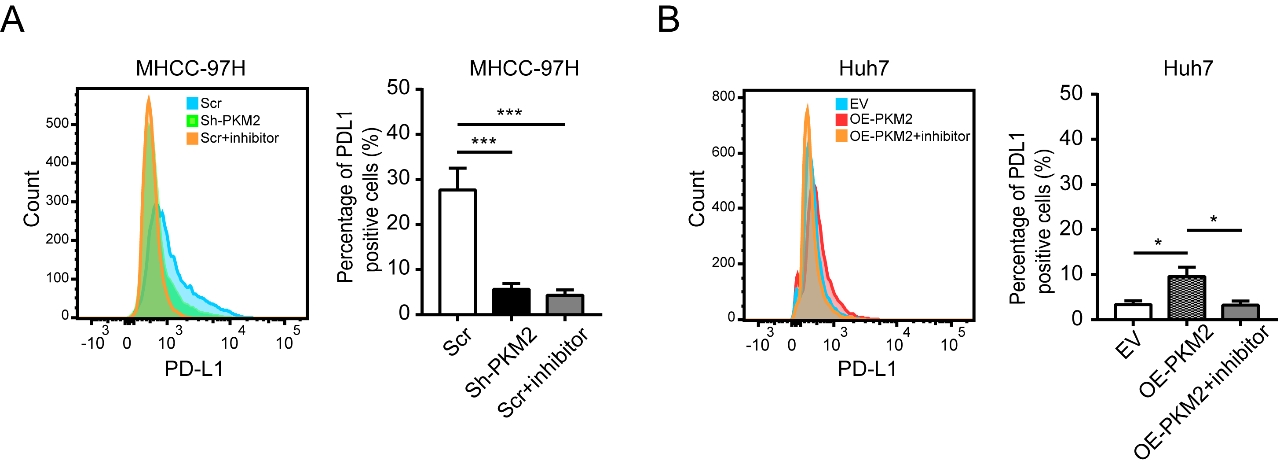


**Supplementary Figure 6.** **PD-L1 expression in PKM2 intervened HCC tumor cells.** (A) Flow cytometric analysis and quantitative analysis of PD-L1 expression in Sh-PKM2 MHCC97-H cells. Left panel: Representative image of flow cytometric analysis. Right panel: quantitative analysis of flow cytometric analysis. (B) Flow cytometric analysis and quantitative analysis of PD-L1 expression in OE-PKM2 Huh7 cells. Left panel: Representative image of flow cytometric analysis. Right panel: quantitative analysis of flow cytometric analysis. n= 5, mean ± SEM, ***P<0.001, **P< 0.01, *P <0.05.
