# Supplementary material for: PKM2 Drives Hepatocellular Carcinoma Progression by Inducing Immunosuppressive Microenvironment
Source: Front Immunol. 2020 Oct 20;11:589997. doi: 10.3389/fimmu.2020.589997 (PMC7606949; doi:10.3389/fimmu.2020.589997)
Supplement: Supplementary file 2 [file Table_2.docx]

Supplementary Tables

**Supplementary Table 1**. Baseline clinicopathological characteristics of HCC patients.

| Characteristics | The cohort in the present study |
| --- | --- |
|  | N=87 |
| Gender |  |
| Female | 14 (16.1%) |
| Male | 73 (83.9%) |
| Age (years) |  |
| ≤50 | 38 (43.7%) |
| >50 | 49 (56.3%) |
| AFP (ng/ml) |  |
| ≤20 | 36 (41.7%) |
| ＞20 | 51 (37.9%) |
| ALT (U/L) |  |
| ≤40 | 54 (62.1%) |
| ＞40 | 33 (37.9%) |
| Tumor size (cm) |  |
| ≤5 | 56 (64.3%) |
| ＞5 | 31 (35.6%) |
| HBV status |  |
| Yes | 68 (78.1%) |
| No | 19 (21.8%) |
| Cirrhosis |  |
| Yes | 70 (80.5%) |
| ` NO | 17 (19.5%) |
| Tumor capsule formation |  |
| Yes | 52 (59.8%) |
| No | 35 (40.2%) |
| Vascular invasion |  |
| Yes | 62 (71.3%) |
| No | 25 (28.7%) |
| Tumor number |  |
| Single | 75 (86.2%) |
| Multiple | 12 (13.8%) |
| Tumor Grade |  |
| 1-2 | 64 (73.6%) |
| 3-4 | 23 (26.4%) |
| BCLC stage |  |
| 0+A | 17 (19.5%) |
| B+C+D | 70 (80.5%) |

HBV, Hepatitis B Virus; AFP, alpha fetoprotein; ALT, alanine aminotransferase; BCLC, Barcelona Clinic Liver Cancer; HBV, hepatitis B virus. *P* <0.05 was considered statistically significant. * Chi-square test.

**Supplementary Table 2**. Relationship between clinicopathological features and PKM2.

| Characteristics | PKM2 expression | | P value |
| --- | --- | --- | --- |
|  | Low (N=35) | High (N=52) |  |
| Gender |  |  |  |
| Female | 7 | 7 | 0.416 |
| Male | 28 | 45 |  |
| Age (years) |  |  |  |
| ≤50 | 11 | 27 | 0.059 |
| >50 | 24 | 25 |  |
| AFP (ng/ml) |  |  |  |
| ≤20 | 15 | 21 | 0.818 |
| ＞20 | 20 | 31 |  |
| ALT (U/L) |  |  |  |
| ≤40 | 24 | 30 | 0.305 |
| ＞40 | 11 | 22 |  |
| Tumor size (cm) |  |  |  |
| ≤5 | 28 | 28 | **0.013*** |
| ＞5 | 7 | 24 |  |
| HBV status |  |  |  |
| Yes | 28 | 40 | 0.733 |
| No | 7 | 12 |  |
| Cirrhosis |  |  |  |
| Yes | 29 | 41 | 0.644 |
| ` NO | 6 | 11 |  |
| Tumor capsule formation |  |  |  |
| Yes | 22 | 30 | 0.630 |
| No | 13 | 22 |  |
| Vascular invasion |  |  |  |
| Yes | 23 | 39 | 0.348 |
| No | 12 | 13 |  |
| Tumor number |  |  |  |
| Single | 32 | 43 | 0.247 |
| Multiple | 3 | 9 |  |
| Tumor Grade |  |  |  |
| 1-2 | 30 | 34 | **0.035*** |
| 3-4 | 5 | 18 |  |
| BCLC stage |  |  |  |
| 0+A | 9 | 8 | 0.233 |
| B+C+D | 26 | 44 |  |

HBV, Hepatitis B Virus; AFP, alpha fetoprotein; ALT, alanine aminotransferase; BCLC, Barcelona Clinic Liver Cancer; HBV, hepatitis B virus; PKM2, Pyruvate kinase M . *P* <0.05 was considered statistically significant. ***** Chi-square test.

**Supplementary Table 3.**  Relationship between clinicopathological features and PD-L1.

| Characteristics | PD-L1 expression | | P value |
| --- | --- | --- | --- |
|  | Low (N=42) | High (N=45) |  |
| Gender |  |  |  |
| Female | 9 | 5 | 0.191 |
| Male | 33 | 40 |  |
| Age (years) |  |  |  |
| ≤50 | 16 | 22 | 0.310 |
| >50 | 26 | 23 |  |
| AFP (ng/ml) |  |  |  |
| ≤20 | 13 | 23 | 0.056 |
| ＞20 | 29 | 22 |  |
| ALT (U/L) |  |  |  |
| ≤40 | 25 | 29 | 0.636 |
| ＞40 | 17 | 16 |  |
| Tumor size (cm) |  |  |  |
| ≤5 | 32 | 24 | **0.026*** |
| ＞5 | 10 | 21 |  |
| HBV status |  |  |  |
| Yes | 31 | 37 | 0.343 |
| No | 11 | 8 |  |
| Cirrhosis |  |  |  |
| Yes | 33 | 37 | 0.668 |
| NO | 9 | 8 |  |
| Tumor capsule formation |  |  |  |
| Yes | 24 | 28 | 0.629 |
| No | 18 | 17 |  |
| Vascular invasion |  |  |  |
| Yes | 29 | 33 | 0.659 |
| No | 13 | 12 |  |
| Tumor number |  |  |  |
| Single | 40 | 35 | **0.018*** |
| Multiple | 2 | 10 |  |
| Tumor Grade |  |  |  |
| 1-2 | 33 | 31 | 0.306 |
| 3-4 | 9 | 14 |  |
| BCLC stage |  |  |  |
| 0+A | 10 | 7 | 0.332 |
| B+C+D | 32 | 38 |  |

HBV, Hepatitis B Virus; AFP, alpha fetoprotein; ALT, alanine aminotransferase; BCLC, Barcelona Clinic Liver Cancer; HBV, hepatitis B virus; PD-L1, programmed cell death ligand 1. *P* <0.05 was considered statistically significant. ***** Chi-square test.

**Supplementary Table1.** Primer sequences used in the study.

| Primer name | Sequence | |
| --- | --- | --- |
|  | Forward | Reverse |
| **Primer for qPCR (Human)** | | |
| PKM2 | AGAACTTGTGCGAGCCTCAA | GAGCAGACCTGCCAGACTC |
| PD-L1 | ATTTGCTGAACGCCCCATAC | TTGGTGGTGGTGGTCTTACC |
| GLUT | GGCCAAGAGTGTGCTAAAGAA | ACAGCGTTGATGCCAGACAG |
| HK2 | GAGCCACCACTCACCCTACT | CCAGGCATTCGGCAATGTG |
| GPI | CAAGGACCGCTTCAACCACTT | CCAGGATGGGTGTGTTTGACC |
| PFKFB2 | TGGGCCTCCTACATGACCAA | CAGTTGAGGTAGCGTGTTAGTTT |
| ALDOA | ATGCCCTACCAATATCCAGCA | GCTCCCAGTGGACTCATCTG |
| GAPDH | GGAGCGAGATCCCTCCAAAAT | GCTCATAAGGACTACCGACTTGG |
| PGAM2 | AGAAGCACCCCTACTACAACTC | TCTGGGGAACAATCTCCTCGT |
| ENO2 | AGCCTCTACGGGCATCTATGA | TTCTCAGTCCCATCCAACTCC |
| PKM1 | CCTTCCAGATCAGGACCTCAG | GGCTGTGATGGGTGGTGAAT |
| LDHA | ACGTGCATTCCCGATTCCTT | AAAGGCTGCCATGTTGGAGA |
| LDHB | CGGGGTAGTACTTGTATGGGG | GCCATTTTGCACAAGGACA |
| β-actin | GGACCTGACTGACTACCTCAT | CGTAGCACAGCTTCTCCTTAAT |
| **Primer for qPCR (Mouse)** | | |
| PKM2 | CATTACCAGCGACCCCACAG | GAGCACTCCTGCCAGACTTG |
| PD-L1 | AATGCTGCCCTTCAGATCAC | ATAACCCTCGGCCTGACATA |
| CD38 | TCTCTAGGAAAGCCCAGATCG | GTCCACACCAGGAGTGAGC |
| CD47 | TGGTGGGAAACTACACTTGCG | CGTGCGGTTTTTCAGCTCTAT |
| CD111 | GACTCCATGTATGGCTTCATCG | CACTCGTTTCTCGTAGGGAGG |
| CD112 | GCATCATTGGAGGTATTATCGCT | GAGGGAGGTCCTTCCAGTTC |
| Siglec15 | CCGAGGCCAGCGTCTACCTGTT | TGATCTAGTCGGCGTCGGGTGG |
| β-actin | gtccctcaccctcccaaaag | gctgcctcaacacctcaaccc |
| **Primer for Sh-PKM2 RNA (Human)** | | |
| Sh1 | CCGGGTTCGGAGGTTTGATGAAATCCTCGAGGATTTCATCAAACCTCCGAACTTTTTTG | AATTCAAAAAAGTTCGGAGGTTTGATGAAATCCTCGAGGATTTCATCAAACCTCCGAAC |
| Sh2 | CCGGGCCCGAGGCTTCTTCAAGAAGCTCGAGCTTCTTGAAGAAGCCTCGGGCTTTTTTG | AATTCAAAAAAGCCCGAGGCTTCTTCAAGAAGCTCGAGCTTCTTGAAGAAGCCTCGGGC |
| **Primer for Sh-PKM2 RNA (Mouse)** | | |
| Sh1 | CCGGATCATTGCCGTGACTCGAAATCTCGAGATTTCGAGTCACGGCAATGATTTTTTG | AATTCAAAAAATCATTGCCGTGACTCGAAATCTCGAGATTTCGAGTCACGGCAATGA |
| Sh2 | CCGGAGATGCTGAAGGAGATGATTACTCGAGTAATCATCTCCTTCAGCATCTTTTTTG | AATTCAAAAAAGATGCTGAAGGAGATGATTACTCGAGTAATCATCTCCTTCAGCATC |
| **Primer for OE-PKM2 (Human)** | | |
|  | ATGTCGAAGCCCCATAGTGA | TCACGGCACAGGAACAACAC |
| **Primer for OE-PKM2 (Mouse)** | | |
|  | ATGCCGAAGCCACACAGTG | TCAAGGTACAGGCACTACACG |
