# Supplementary material for: PKM2 Drives Hepatocellular Carcinoma Progression by Inducing Immunosuppressive Microenvironment
Source: Front Immunol. 2020 Oct 20;11:589997. doi: 10.3389/fimmu.2020.589997 (PMC7606949; doi:10.3389/fimmu.2020.589997)
Supplement: Supplementary file 3 [file Table_3.docx]

Supplementary Materials and Methods

**Plasmid and cell transfections**

To establish stable PKM2 knockdown cell lines, short hairpin RNA (shRNA) sequences against the PKM2 (Human) sequence (sh-PKM2-1: GTTCGGAGGTTTGATGAAATC; sh-PKM2-2: GCCCGAGGCTTCTTCAAGAAG) and PKM2 (Mouse) sequence (sh-PKM2-1: ATCATTGCCGTGACTCGAAAT; sh-PKM2-2: AGATGCTGAAGGAGATGATTA) were cloned into the pLKO.1 puro retroviral vector. The recombinant vectors were co-transfected into HEK293T cells with GAG-POL and VSVG plasmid using Lipofectamine 2000 (Invitrogen, MA, USA). For the PKM2 overexpression stable cell lines, PKM2 cDNA (Human: NP_002645.3) and (Mouse: NP_001365797.1) was cloned into pCDH-CMV-MCS-EF1-puro vector. The recombinant vector was used to package pseudoviral particles with two other packaging plasmids psPAX2 and pMD2.G by co-transfecting into HEK293T cells.

**Cell proliferation assay**

Cell proliferation assay was carried out by using Cell Counting Kit-8 (Dojindo, Kumamoto, Japan). Cancer cells were seeded into 96-well plates at a density of 3×10^3^/100μl cells per well (n = 5 for each time point) and incubated for 12, 24, 36, 48, 60 and 72 h. Then the medium was replaced with 10μl CCK-8 solution and 90μl complete medium. The absorbance at 450 nm was measured after incubation for 1h at 37°C in 5% CO_2_.

**Colony formation assay**

Cancer cells were seeded in six-well plates at a density of 1 × 10^3^ cells/well and incubated at 37°C in 5% CO_2_ for 2 weeks. Then cells were fixed in 4% formalin for 20 min and stained with 1% crystal violet for 20 min. The number of colonies was counted.

**Migration and invasion assays**

To examine the abilities of migration and invasion of HCC cells, 24-well Transwell chambers, with upper and lower culture compartments, separated by polycarbonate membranes with 8 µm pores (BD Pharmingen) were used. The bottom chamber was filled with DMEM medium supplemented with 20% FBS. 1.5 × 10^4^ cells without Matrigel were prepared for migration, and 2.5 × 10^4^ cells with Matrigel were prepared for invasion 1 hour after seeding 100μl Matrigel in the upper chamber. The cells suspended in serum-free medium were then seeded into the upper chamber and maintained in normal cell culture condition mentioned above. Cells that migrated or invaded through the membrane were stained with crystal violet, and calculated with light microscope.

**Survival analysis and correlation analysis using the GEPIA web tool**

The online database Gene Expression Profiling Interactive Analysis (GEPIA, http://gepia.cancer-pku.cn/) was used to analyze the RNA sequencing expression data related to our project based on The Cancer Genome Atlas (TCGA) and the Genotype-Tissue Expression (GTEx) projects^1^. GEPIA performs survival analyses based on gene expression levels, and uses a log-rank test for hypothesis evaluation. GEPIA plots gene expression by pathological stages based on the TCGA clinical annotation. GEPIA performs a pairwise gene correlation analysis for any given set of TCGA and/or GTEx expression data using Pearson correlation statistics.

**Tissue microarrays (TMA) and immunohistochemistry (IHC) of HCC tissues**

We applied IHC staining analysis on a cohort of 87 HCC patients. Clinical samples from patients were obtained after acquiring their consent in accordance with the protocol approved by the Ethics Boards of Huashan Hospital, Fudan University (Shanghai, China).

Formalin-fixed and paraffin-embedded tissues were used to construct TMA as previously described^2^. Briefly, two four-micron core biopsies from the donor blocks were taken and transferred to the recipient paraffin block at predefined array positions and constructed 87 cases of TMA blocks in this study.

IHC staining was performed as described previously**^3^**. Briefly, following deparaffinization, rehydrating and antigen retrieval, primary antibodies were applied to slides, incubated at 4℃ overnight, followed by incubation with secondary antibody (Dako Denmark A/S, Glostrup, Denmark) at 37℃ for 30 minutes. Anti-PKM2 antibody (Proteintech, 1:50), anti-PD-L1 antibody (Abcam, 1:50) and Ki67 (Abcam, 1:50) were used as primary antibodies. Staining was carried out with Diaminobenzidine (DAB) and counterstaining was performed with hematoxylin. TUNEL assay kit (Abcam) was used to detect apoptosis of tumor tissues.

Scoring for PKM2 and PD-L1 staining was conducted using percentage score × staining intensity score. The percentage of positive-staining cells: 0-5% scored 0, 6-25% scored 1, 26-50% scored 2, 51-75% scored 3 and more than 75% scored 4; staining intensity: no staining scored 0, weakly staining scored 1, moderately staining scored 2 and strongly staining scored 3.

**Western blot**

Western blotting was carried out as described before^2,4^. Total protein was extracted by RIPA buffer containing protease cocktail inhibitor. Protein samples were separated by sodium dodecyl sulfate polyacrylamide gel electrophoresis (SDS-PAGE) and then transferred onto polyvinylidene fluoride (PVDF) membranes. After blocking with 5% skim milk in TBS-T (BD Pharmingen), PVDF membranes were incubated with the primary antibody overnight at 4°C and then with the secondary antibody for 1h at room temperature. The following antibodies were used: anti-PKM2 (Proteintech,1:1000), anti-PD-L1 (Proteintech, 1:1000), anti-β-Tubulin (Abclonal, 1:1000). Protein bands were detected by image acquisition using ImageQuant^TM^ LAS 4000 (GE Healthcare Life Sciences).

**RNA isolation and real time quantitative reverse transcription PCR (qRT-PCR)**

RNA isolation and cDNA obtaining were performed as described previously^2,4^. For qRT-PCR analysis, cDNA was amplified using SYBR Green Realtime PCR Master Mix (Takara, Japan). qRT-PCR reactions were performed in triplicates as the following conditions: 95°C/20s, 40 cycles of 95°C/60s and 60°C/20s using the ABI PRISM 7900 Sequence Detection System (Applied Biosystems, Foster City, CA, USA) and repeated at least three times. Relative mRNA levels were analyzed by the -ΔΔCt method using β-actin as the endogenous control and presented as 2^^-ΔΔCt^. All the primers are listed in **Supplementary Table 4**.

**Lactate detection assay**

Lactate production of HCC cells was quantified using the BioVision Lactate Assay Kit (Bio Vision, CA, USA) as described previously^5^. The cell culture medium was collected from the treated cells and added to lactate assay buffer. The reaction was incubated for 30mins at room temperature, and absorbance was measured at 570nm using a microplate reader.

**T cell mediated cytotoxicity assay**

Mouse HCC cells including Sh-Ctrl Hepa1-6, Sh-PKM2 Hepa1-6, Control Hepa1-6 and OE-PKM2 Hepa1-6 (1 × 10^4^ cells/per well) were seeded in 12-well plate, which were pretreated with anti-CD3 antibody (5μg/ml) (BD Biosciences) at 4°C for 12h, and incubated at 37°C with 5% CO_2_ for 12h. CD8^+^ T cells were isolated from the spleen, using positive isolation microbeads (Miltenyi Biotec), and were co-cultured with cancer cells in RPIM 1640 medium (Gibco) supplemented with anti-CD28 antibody (5μg/ml) (BD Biosciences) at 37°C with 5% CO_2_ for 4 days. Then the surviving tumor cells were visualized using crystal violet staining. Each experiment was repeated five times.

**Flow cytometry**

The single cells were isolated from mouse subcutaneous xenograft tumor by a semi-automated combined mechanical/enzymatic process using the gentleMACS Dissociator and the Tumor Dissociation Kit (mouse, Miltenyi Biotec, Germany). ACK lysis buffer was used to lyse red blood cells. Cell suspensions were blocked with mouse FcR blocking reagent (Miltenyi Biotec, USA) and then stained with antibodies against mouse CD3, CD4, CD8, CD45, CD279, IFN-γ and matched isotype controls, depending on the experiment. For IFN-γ staining, single cells were restimulated with 1μl/ml Cell Activation Cocktail (with Brefeldin) in 5% CO_2_ before intracellular staining at 37°C for 8h. These antibodies were obtained from eBioscience; PE/cy7 anti-mouse CD3 antibody, APC anti-mouse CD45 antibody, Percp/cy5.5 anti-mouse CD4 antibody, FITC anti-mouse CD8 antibody, PE anti-mouse CD279 antibody, PE anti-human CD279 antibody. Samples were run on a BD FACSVerse™ (BD Biosciences, USA) and analyzed using FlowJo software (TreeStar, USA).

**Statistical analysis**

Statistical analyses were carried out using Statistical Package for Social Sciences Version 21.0 (SPSS 21.0) and Graphpad Prism 7.0. The analysis of variance (ANOVA) test was used to compare mean values among three or more groups, whereas independent-sample two-sided Student’s test was used to compare two groups with normal distribution data. The correlation analysis was determined by Pearson. Kaplan–Meier survival analyses were used to estimate the overall survival and disease-free survival, and the log-rank test was used to assess the differences. All statistics were two sided and p<0.05 was considered statistically significant.

**REFERENCE**

1 Tang Z, Li C, Kang B, Gao G, Li C & Zhang Z. GEPIA: a web server for cancer and normal gene expression profiling and interactive analyses. *Nucleic Acids Res* (2017) 45, W98-w102. doi:10.1093/nar/gkx247

2 Zhang Z, Li TE, Chen M, Xu D, Zhu Y, Hu BY *et al.* MFN1-dependent alteration of mitochondrial dynamics drives hepatocellular carcinoma metastasis by glucose metabolic reprogramming. *Br J Cancer* (2020) 122, 209-220. doi:10.1038/s41416-019-0658-4

3 Zhu Y, Wang XY, Zhang Y, Xu D, Dong J, Zhang Z *et al.* Programmed death ligand 1 expression in human intrahepatic cholangiocarcinoma and its association with prognosis and CD8(+) T-cell immune responses. *Cancer management and research* (2018) 10, 4113-4123. doi:10.2147/cmar.s172719

4 Ye QH, Zhu WW, Zhang JB, Qin Y, Lu M, Lin GL *et al.* GOLM1 Modulates EGFR/RTK Cell-Surface Recycling to Drive Hepatocellular Carcinoma Metastasis. *Cancer Cell* (2016) 30, 444-458. doi:10.1016/j.ccell.2016.07.017

5 Li H, Li CW, Li X, Ding Q, Guo L, Liu S *et al.* MET Inhibitors Promote Liver Tumor Evasion of the Immune Response by Stabilizing PDL1. *Gastroenterology* (2019) 156, 1849-1861.e1813. doi:10.1053/j.gastro.2019.01.252
